# Hyperspectral indices data fusion-based machine learning enhanced by MRMR algorithm for estimating maize chlorophyll content

**DOI:** 10.3389/fpls.2024.1419316

**Published:** 2024-10-16

**Authors:** Attila Nagy, Andrea Szabó, Ahmed Elbeltagi, Gift Siphiwe Nxumalo, Erika Budayné Bódi, János Tamás

**Affiliations:** ^1^ Institute of Water and Environmental Management, Faculty of Agricultural and Food Sciences and Environmental Management, University of Debrecen, Debrecen, Hungary; ^2^ National Laboratory for Water Science and Water Safety, Institute of Water and Environmental Management, Faculty of Agricultural and Food Sciences and Environmental Management, University of Debrecen, Debrecen, Hungary; ^3^ Agricultural Engineering Dept., Faculty of Agriculture, Mansoura University, Mansoura, Egypt

**Keywords:** maize chlorophyll estimation, hyperspectral indices, minimum redundancy maximum relevance (MRMR) algorithm, spectral bands, machine learning

## Abstract

Accurate estimation of chlorophyll is essential for monitoring maize health and growth, for which hyperspectral imaging provides rich data. In this context, this paper presents an innovative method to estimate maize chlorophyll by combining hyperspectral indices and advanced machine learning models. The methodology of this study focuses on the development of machine learning models using proprietary hyperspectral indices to estimate corn chlorophyll content. Six advanced machine learning models were used, including robust linear stepwise regression, support vector machines (SVM), fine Gaussian SVM, Matern 5/2 Gaussian stepwise regression, and three-layer neural network. The MRMR algorithm was integrated into the process to improve feature selection by identifying the most informative spectral bands, thereby reducing data redundancy and improving model performance. The results showed significant differences in the performance of the six machine learning models applied to chlorophyll estimation. Among the models, the Matern 5/2 Gaussian process regression model showed the highest prediction accuracy. The model achieved R^2^ = 0.71 for the training set, RMSE = 338.46 µg/g and MAE = 264.30 µg/g. In the case of the validation set, the Matern 5/2 Gaussian process regression model further improved its performance, reaching R^2^ =0.79, RMSE=296.37 µg/g, MAE=237.12 µg/g. These metrics show that Matern’s 5/2 Gaussian process regression model combined with the MRMR algorithm to select optimal traits is highly effective in predicting corn chlorophyll content. This research has important implications for precision agriculture, particularly for real-time monitoring and management of crop health. Accurate estimation of chlorophyll allows farmers to take timely and targeted action.

## 1 Introduction

Maize (*Zea mays* L.) is the global second-largest arable crop, playing a pivotal role in global agriculture due to its status as a staple food, animal feed, and industrial crop (Grote et al., 2021). Its adaptability, versatility, and widespread cultivation contribute significantly to initiatives addressing food security and economic growth in developing countries. Maize is high in starch content, typically around 65%. With starch as its primary carbohydrate, maize serves as a major energy source for both humans and animals, making it a critical component of the global food supply. Given its significance, monitoring the health and nutrient status of maize is essential for optimizing yield and ensuring sustainable agricultural practices. Maize, like other green plants, exhibits distinct absorption maxima in the blue (around 430 nm) and red (approximately 660 nm) regions of the spectrum due to chlorophyll, while reflecting green light (around 550 nm), which gives maize leaves their characteristic color (Al-Abbas et al., 1974; Weber et al., 2012). The near-infrared spectrum (700-1300 nm), where maize shows high reflectance, is particularly useful in assessing plant health, as it is influenced by the internal structure of leaves (Nagy and Tamás, 2013; Szabó et al., 2019). Monitoring these spectral properties allows for the detection of water stress, chlorophyll content, and overall plant health, providing critical insights for crop management. Leaf colour is determined by various pigments like anthocyanin, xanthophyll, carotenoid, and chlorophyll, all of which play a crucial role in light conversion and energy utilization (Winkel-Shirley, 2002). In addition to the initial harm caused by oxidative stress impacting lipids, proteins, nucleic acids, and chlorophyll breakdown, plants undergoing drought stress undergo subsequent damage. During severe drought conditions, chlorophyll a and b levels decline (Sircelj et al., 2007), and analysing chlorophyll levels proves valuable in studying how plants respond to biotic stress (Ghobadi et al., 2013; Neto et al., 2017). The concentration of chlorophyll is closely linked to the nitrogen content of plants and, as a result, is intricately associated with the photosynthesis process. Usha and Singh (2013) illustrated that the sensitivity of reflectance to stress-induced chlorophyll content is particularly high in the 690-700 nm range. If stress possesses sufficient potency to impede the formation of chlorophyll, heightened reflectance is initially detected at the typical absorption wavelengths. The 760-790 nm range proves effective for determining plant water stress (Jung, 2005; Nemeskéri et al., 2009). Additionally, Yu et al. (2014) noted that wavelengths at 730 and 960 nm are linked to water absorption bands. Maize plants, like other green plants, have absorption maxima in the blue (about 430 nm) and red (approximately 660 nm) sections of the spectrum owing to chlorophyll absorption. This reflectance relates to light scattering by leaf interior structures and can be an indicator of plant health (Al-Abbas et al., 1974; Weber et al., 2012). Current methods for field maize chlorophyll estimation face limitations such as the destructiveness and labor-intensity of traditional sampling, variability and calibration challenges in non-destructive optical sensors, and the high costs and complexity of advanced remote sensing techniques (Fu and Jiang, 2022). Environmental factors and temporal variability further complicate accurate assessments, while scalability and practical application remain concerns, especially for large-scale operations (Bramer et al., 2018). Spectroscopic technology has gained attention for its ability to monitor plant growth and nutrient status non-destructively, offering a time-efficient, cost-effective, and detailed analysis of plant health (Qiao et al., 2020). In contrast, spectroscopic techniques, particularly those involving visible-near infrared (Vis-NIR) detection, allow for real-time, non-invasive estimation of chlorophyll levels by identifying specific wavelengths that correlate with chlorophyll absorption and reflectance characteristics (Chen et al., 2020).

In the spectroscopic determination of chlorophyll content, various machine-learning techniques are employed to formulate accurate predictions by establishing correlations between input spectral data and the associated chlorophyll content. Combining hyperspectral indices with machine learning models (MLMs) can offer superior performance over traditional methods by leveraging the high-dimensional data from hyperspectral imaging and the advanced analytical capabilities of MLMs. Hyperspectral indices provide detailed spectral information, capturing subtle variations in chlorophyll content and plant health, while MLMs can analyze complex patterns and relationships within this data to improve accuracy and prediction. This integration enhances the ability to detect and quantify chlorophyll levels more precisely and dynamically, leading to more informed and effective agricultural nutrient and water management (Yoosefzadeh-Najafabadi et al., 2021). Among these techniques, the Minimum Redundancy Maximum Relevance (MRMR) algorithm emerges as a prominent feature selection approach widely adopted in both machine learning and bioinformatics (Radovic et al., 2017; Ding and Peng, 2005). The primary objective of the MRMR algorithm is to identify a subset of features (variables) from an extensive set, emphasizing both the relevance of these features to the target variable and the minimization of redundancy among the selected features. Recognized for its efficacy, the MRMR algorithm proves instrumental in enhancing the selection of the most informative spectral bands or indices crucial for precise chlorophyll estimation (Li et al., 2018). Its application ensures a refined set of features that optimally contribute to the accuracy and reliability of chlorophyll content predictions, thus elevating the overall effectiveness of machine learning models in this spectroscopic context. Robust linear (RL) regression is applied to improve the accuracy of chlorophyll content predictions by mitigating the impact of outliers in the remote sensing data, hyperspectral data, multi-sensor data fusion, drought or stress conditions, and longitudinal studies (Park et al., 2015; Luo et al., 2019; Liu et al., 2019). In field spectroscopy, stepwise regression is applied to build models that predict chlorophyll content using a subset of relevant spectral features. Stepwise regression is a useful tool for variable selection to adapt or integrate chlorophyll detection models to changing environmental conditions or stress factors and cross-sensor calibration (Liu et al., 2018; Song et al., 2021). Support Vector Machines (SVM) is employed as a supervised learning algorithm to develop models that can classify or estimate chlorophyll content based on spectral data. It is effective in scenarios where the relationship between spectral features and chlorophyll content is complex or non-linear, such as diagnosing citrus greening disease and nutritional stress in citrus leaves (Cen et al., 2017). When the spectral data is high-dimensional and exhibits non-linear patterns, A Fine Gaussian SVM, a type of Support Vector Machine (SVM) model that utilizes a Gaussian kernel, is highly effective in classifying leaves and plants as either disease-free or infected (Lacotte et al., 2022; Jia et al., 2023). The Matérn 5/2 kernel Gaussian process regression specific form of covariance function commonly used in Gaussian Process (GP) regression. It provides a flexible and smooth representation of the underlying relationship between spectral features and chlorophyll content. It has been utilized to forecast the acoustic performance of composite materials made from agricultural crop waste (Puyana-Romero et al., 2022). It is based on a methodology used to optimize hyperparameters of noisy, expanding black-box functions that represent a systematic approach to modeling uncertainty. The method performs better in detecting tea leaf chlorophyll and optimization of functions (Sonobe et al., 2020; Snoek et al., 2015). A trilayered neural network involves neural network architecture with three tiers of nodes which include an input layer, a concealed layer, and an output layer. It is applied to chlorophyll detection for yield predictions (Lagrazon and Tan, 2023), precision agriculture (Zahra et al., 2023), and soil health monitoring (Ropelewska et al., 2023). This type of neural network can learn intricate patterns in the data for accurate predictions. As chlorophyll is an indicator of plant vigor, it is, therefore, essential to provide spatially resolved real-time or near real-time monitoring of chlorophyll levels without damaging the plant or disrupting its environment. Existing methods often lack standardization and effective integration with machine learning models, limiting their reliability and broader applicability (Maier, 2021).

The primary aim of the study is to expand the area of remote sensing in agricultural monitoring using proximal sensors by creating a new rapid non-invasive approach for predicting crop chlorophyll content with hyperspectral data, machine learning models, and MRMR feature selection technique. The specific objectives are (1) to develop and test novel proximal sensor-based spectral indices that cover a broader range of wavelengths; (2) to assess the precision of six machine learning algorithms which are Robust Linear (RL), Stepwise Regression (SR), Support Vector Machines (SVMs), Fine Gaussian SVM (FG-SVM), Matern 5/2 Gaussian Process Regression (MGPR) and Trilayered Neural Networks (TNN) in predicting chlorophyll levels in maize leaves, thereby enhancing the ability to detect and quantify chlorophyll content with higher precision. The study seeks to close the gap where chlorophyll estimations are generally not plant-specific by offering an integrated and refined approach to improve reliability and accessibility in chlorophyll estimation. The ultimate objective is to give farmers and agricultural stakeholders new approaches for more precise and dependable tools for measuring crop health while promoting sustainability, efficiency, and scalability in crop management practices.

## 2 Materials and methods

### 2.1 Study site

The study site is in the Pannonian region on the Northern Great Plain, marked by coordinates (latitude: 47°48’18.60”N, longitude: 22°9’43.89”E, altitude: 144 m), at the boundary of a climate zone characterized by moderately warm and cold (continental). This location is an alluvial cone plain primarily covered with sand. Covering an expanse of 87.5 hectares, the area is designated as irrigated arable land and is equipped with a linear irrigation system. Due to past melioration and drainage activities conducted in the previous century, the active water network is currently sparse, and the landscape experiences low horizontal fragmentation. The European Commission declared the study site a nitrate-vulnerable area in 2010. Over the past ten years, the annual sunshine hours have varied between 1900 and 2000, with 800 hours during the summer season and 170 hours in winter, according to the Hungarian National Weather Service (HNWS) (2020). The average annual temperature falls within the range of 9.6°C to 12.6°C. In summer, the daily maximum temperature can surpass 34°C, while winter typically sees minimum temperatures below -17.0°C. The annual rainfall is recorded at 570-600 mm, with approximately 350-360 mm occurring during the summer season. The prevailing wind directions are from the northeast and southeast, with an average speed of 2.5 m/s (Magyar et al., 2023).

**Figure 1:**
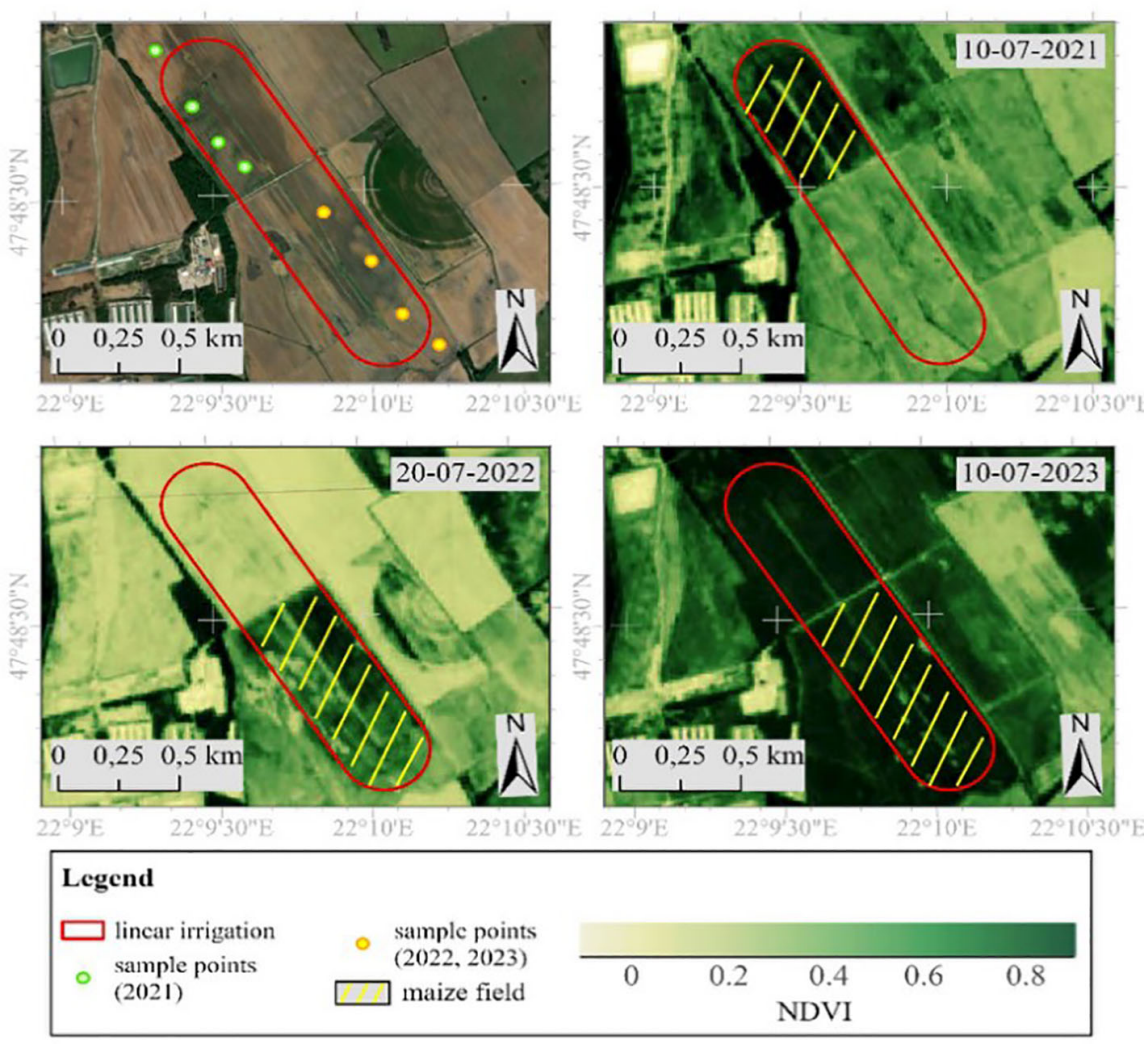
Study site.

In 2021, maize P0725 - FAO 580 was sown on 13.05.2021 and FAO 530 on 21.05.2021 at 76 000 grains/ha and 76.2 cm row spacing. Harvest time was 22-23.09.2021 with an average yield of 34.14 t/ha at ~35% dry matter. In 2022, the sowing date was 19.04.2022 with the maize variety PIONEER P0725 at a grain density of 76 000 grains/ha and a row spacing of 75 cm. The harvest date was 23.08.2022, with an average yield of 34.40 t/ha. In 2023, the maize variety RAAGT Mexxpledge was sown on 29.04.2023 at a grain density of 72 000 grains/ha and a row spacing of 75 cm. The harvest date was 16.08.2022. The silage yield of the irrigated area was 40.4 t/h, and the yield of the non-irrigated area was 32.8 t/ha. Maize was grown in sandy soils with extreme water balance, which poses a particularly high risk of water deficit and heat stress (Magyar et al., 2023). To ensure homogeneity of sampling, leaf samples were taken from the upper biomass level of selected maize plants to determine pigment content (**Figure 1**).

### 2.2 Measurement method and data processing

Sampling was carried out 9 times (3/year) in 2021, 2022, and 2023 at 5 sampling areas, during which a total of 540 samples were collected from irrigated and non-irrigated areas. One sampling area was from an unirrigated part of the field, and the rest four were from irrigated parts. The selection of sampling areas at the irrigated part was based on different soil physical parameters (Magyar et al., 2023). The sampling areas were selected based on sampling was carried out between 10-12 h, for which 12 samples were taken from each selected point. Leaf samples were measured in the laboratory within 6 h after storage and transport, chilled at 4°C. Samples were destructed with 80% acetone and 1 g quartz sand for homogeneity. After extraction, the suspensions were centrifuged at 3000 rpm for 3 min and the clear solution was transferred to a 2,5 ml cuvette. The absorbance of the solution was measured with a spectrophotometer (SECOMAN Anthelie Light II) at 470 nm, 644 nm, and 663 nm.

The chlorophyll content of the samples was determined using the equation published by Droppa et al. (2003) (Equation 1):


(1) $$\begin{array}{c} \text{Chlorophyll }\left(\text{a}+\text{b}\right)\;\text{μg}/\text{g fresh weight}\\ \;=\left(20,2*{\text{A}}_{644\text{nm}}+8,02*{\text{A}}_{663\text{nm}}\right)*\text{V}/\text{w} \end{array}$$

where:

V = volume of tissue extract (ml)

w = fresh weight of tissue (g)

A = absorbance

The AvaSpec 2048 spectrometer was used to collect spectral data from leaf samples in the wavelength range 400-1000 nm with an accuracy of 0.6 nm. The system consists of a spectrometer, an AvaLightHAL halogen light source, and a special patented sampling box, which performs measurements in the dark thus making the measurements free from external light and various noises. Changes in light conditions (e.g. LED or fluorescent tube) affects the reflectances at specific wavelegthses, therefore elimination of these background light is essential for proper measurements. After calibration of the spectrometer to a white and dark reference, the leaf sample was placed under the illumination of the spectrometer and measurements were performed in three replicates (Szabó et al., 2019).

### 2.3 Model building and performance assessment

The results were subjected to statistical analysis using SPSS software, using PCA with Varimax rotation to compress the data, detect outliers, and reveal patterns and internal structure within the overall data set. The aim of this approach was to identify the wavelengths with the largest variation in factor weights. Since the major changes in leaf samples were in the pigments, especially chlorophyll, the highest variation in reflectace is possibly due to the different chlorophyll contents in leaves making PCA results optimal for identifying wavelengths sensitive to plant chlorophyll content (Liu et al., 2017). Varimax rotation, known for generating separate factor loadings, facilitated the assignment of individual objects to a single factor (Jolliffe, 2002). Varimax rotation, an orthogonal rotation method, was specifically selected to make the output of PCA more interpretable by maximizing the variance of the squared loadings of each component. This results in more distinct and meaningful patterns, making it easier to identify and analyze specific features within the hyperspectral data, ultimately enhancing the clarity and reliability of the analysis in this context. In addition to PCA, the standard deviation (SD) of spectral features was also examined to identify the wavelengths with the highest variability, indicating potential chlorophyll changes. Using standard deviation (SD) to examine the wavelengths of spectral data is a simple method to identify regions of high variability. These regions are often of interest because high variability can indicate significant differences in the underlying samples that may be related to key characteristics or chemical components present in the samples. By identifying a wavelength with a high SD, we can focus on specific parts of the spectrum that may contain the most informative features. This pre-processing step helps to reduce the dimensionality of the data even before more complex methods such as PCA are applied, by potentially excluding low variability wavelengths that are unlikely to contribute meaningful information to the analysis. Following the selection of chlorophyll sensitive wavelengths in the 400-1000 nm range, spectral indices were created using the most and least sensitive wavelengths based on PCA and SD results, with the combined results increasing the likelihood of capturing the most important spectral features in the analysis. Alongside the developed models, an existing and widely used Vegetation Index (VI) was computed for comparative analysis. The Normalized Difference Vegetation Index (NDVI) (Equation 2) and the Red Edge Position (REP) (Equation 3) index were determined by identifying the maximum slope point in the plant leaf reflectance spectrum between red and near-infrared wavelengths. The Red Edge Normalized Vegetation Index (NDVI_705_) (Equation 4) (Potter et al., 2012) was introduced as a modification, focusing on a narrower waveband at the chlorophyll absorption edge (e.g., 705 nm) (Sims and Gamon, 2002; Moroni et al., 2013). This modification, more influenced by chlorophyll content, finds applications in precision agriculture, forest monitoring, fire detection, and vegetation stress assessment (Cundill et al., 2015). The Modified Red Edge Simple Ratio Index (Equation 5), utilizing red edge bands with a correction for mirror reflection, serves purposes in precision agriculture, forest monitoring, and vegetation stress detection. The Modified Red Edge Normalized Difference Vegetation Index (MNDVI) is a tool developed for analyzing vegetation health and structure through satellite imagery or remote sensing data. Unlike its predecessor, the Normalized Difference Vegetation Index (NDVI), which compares near-infrared (NIR) and red bands, MNDVI leverages the red-edge band. Positioned between visible red and near-infrared bands, the red-edge band is particularly attuned to nuanced alterations in vegetation conditions. This adjustment enhances MNDVI’s applicability in diverse fields like precision agriculture and ecological surveillance (Equation 6). The Photochemical Reflectance Index (PRI) (Equation 7) responds to changes in carotenoid pigments in living foliage, indicating photosynthetic light utilization efficiency. PRI, valuable for assessing vegetation reactions to stress, can be combined with satellite data or remote sensing techniques to evaluate overall ecosystem health. Unlike other indices, PRI captures dynamic physiological changes that can affect chlorophyll content, particularly under varying environmental conditions (Filella and Peñuelas, 1994; Gamon et al., 1997). When used with other indices, PRI provides a complementary perspective. While these other indices primarily focus on static measurements of chlorophyll or structural attributes, PRI adds a dynamic component by reflecting short-term changes in photosynthetic activity, thereby offering a more comprehensive view of plant health and chlorophyll content (Gitelson et al., 1996; Sims and Gamon, 2002) Lastly, the Modified Chlorophyll Absorption Ratio Index (MCARI) (Equation 8) is responsive to leaf chlorophyll concentration and soil reflectance. High MCARI values generally indicate low leaf chlorophyll content, requiring interpretation in conjunction with NDVI or Leaf Area Index (LAI) for comprehensive analysis (Nagler et al., 2000). The Vogelmann Red-Edge Index 2 (VREI2) (Equation 9) is a special index for monitoring land use areas and crops, especially in agricultural applications. VREI2 is a spectral index that uses two near-infrared bands and one red band from sensor readings (Vogelmann et al., 1993). Overall, indices like PRI, REP, and MNDVI outperform NDVI in scenarios requiring precise monitoring of chlorophyll content and early detection of plant stress. Their practical applications in agriculture include optimizing resource use, enhancing crop management strategies, and improving yield predictions (Negrisoli et al., 2022).


(2) $$\begin{array}{l} \text{Normalized Difference }\frac{NIR-R}{NIR+R}\\ \text{Vegetation Index} \end{array}$$

(3) $$\text{Red Edge Position}=700+40\frac{\left(\lambda_{670}+\lambda_{780}\right)/2-\lambda_{700}}{\lambda_{740}-\lambda_{700}}$$

(4) $$\begin{array}{l} \text{Red Edge Normalized }\frac{\lambda_{750}-\lambda_{705}}{\lambda_{750}+\lambda_{705}}\\ \text{Difference Vegetation Index} \end{array}$$

(5) $$\begin{array}{l} \text{Modified Red Edge Simple }\frac{\lambda_{750}-\lambda_{445}}{\lambda_{705}+\lambda_{445}}\\ \text{Ratio Index} \end{array}$$

(6) $$\begin{array}{l} \text{Modified Red Edge Normalized }\frac{\lambda_{750}-\lambda_{705}}{\lambda_{750}+\lambda_{705}-2\lambda_{445}}\\ \text{Difference Vegetation Index} \end{array}$$

(7) $$\begin{array}{l} \text{Photochemical Reflectance }\frac{\lambda_{531}-\lambda_{570}}{\lambda_{531}+\lambda_{570}}\\ \text{Index} \end{array}$$

(8) $$\begin{array}{l} \text{Modified Chlorophyll }\left[\left(\lambda_{700}-\lambda_{670}\right)-0,2\left(\lambda_{700}-\lambda_{550}\right)\right]*\left(\frac{\lambda_{700}}{\lambda_{670}}\right)\\ \text{Absorption Ratio Index} \end{array}$$

(9) Vogelmann red-edge index 2 λ757−λ720λ757+λ720

The simple linear regression method was used to generate models to estimate chlorophyll content. The coefficient of determination (R^2^) (Equation 10) was used to compare the strength of the regression models. For validation every third data, overall 180 independent samples were used. In the case of ML model, training and testing datasets were sorted randomly before training and testing the developed models, after cleaning process to guarantee evaluation of the models performance and accuracy without any personal intervention. To measure the accuracy of the predictive models Root Mean Square Error (RMSE) (Equation 11), Mean Absolute Error (MAE) (Equation 12), Mean Bias Deviation (MBD) (Equation 13) and Mean Squared Prediction Error (MSPE) (Equation 14).


(10) $${\text{ R}}^{2}=1-\;\frac{\sum_{\text{i}=1}^{\text{N}}{\left(y_{i}-\overset{´}{y_{i}}\right)}^{2}}{\sum_{\text{i}=1}^{\text{N}}{\left(y_{i}-\bar{y}\right)}^{2}}$$

(11) $$RMSE=\sqrt{\frac{\sum_{i=1}^{n}{\left(y_{i}-\overset{´}{y_{i}}\right)}^{2}}{n}}$$

(12) $$\text{MAE}=\;\frac{1}{n}\sum_{\text{i}=1}^{\text{N}}\left|\overset{´}{{\text{y}}_{i}}-\text{yi}\right|$$

(13) $$\text{MBD}=\;\frac{1}{n}\sum_{\text{i}=1}^{\text{N}}\left(\text{yi}-\overset{´}{{\text{y}}_{I}}\right)$$

(14) $$\text{MSPE}=\;\frac{1}{n}{\sum_{\text{i}=1}^{\text{N}}\left(\text{yi}-\overset{´}{{\text{y}}_{I}}\right)}^{2}$$

where:

y_i_: estimated value;

$\overset{´}{{\text{y}}_{I}}$ : measured value;

$\bar{y}$ : mean value of reference samples

n: number of samples used for validation.

### 2.4 Machine learning models for estimating chlorophyll content in plants

Multicollinearity refers to a situation in which two or more predictor variables in a regression model are highly correlated with each other, making it difficult to determine the individual effect of each predictor on the dependent variable (Peng et al., 2005). This means that the predictors carry overlapping information about the target variable. If the predictors are highly correlated, the variance of the estimated regression coefficients increases. This inflation makes the coefficients unstable, causing them to fluctuate significantly for small changes in the data. Models affected by multicollinearity are more prone to overfitting when the model captures noise instead of the underlying relationship in the data. Although the model may fit well on training data, it may degrade performance on unseen data, leading to poor generalization. The instability of the coefficient estimates in the presence of multicollinearity can make the model’s predictions unreliable. This can cause problems in interpreting coefficients, standard errors and overall model performance. To detect multicollinearity, the Variance Inflation Factor (VIF) was calculated. The VIF measures how much the variance of the regression coefficient is inflated due to multicollinearity. The tolerance, which is the reciprocal of the VIF, was also calculated. VIF values above 5 and tolerance values below 0.1 are often taken as an indication of multicollinearity (Forthofer et al., 2007).

#### 2.4.1 The minimum redundancy maximum relevance algorithm

MRMR is a feature selection method designed to handle the challenges posed by multicollinearity effectively. MRMR explicitly selects features that are minimally redundant with each other. By focusing on reducing redundancy, MRMR ensures that the selected features provide unique information. This directly addresses the problem of multicollinearity, where highly correlated predictors carry overlapping information. By minimizing redundancy, MRMR helps to mitigate the instability and inflated variances that multicollinearity introduces in a model. This dual focus on relevance and redundancy helps build a more interpretable and effective model. By selecting a subset of features that are both relevant and non-redundant, MRMR contributes to the stability of the model’s coefficients. This stability is crucial in producing reliable predictions and avoiding the overfitting associated with multicollinearity. A stable model with independent features is more likely to generalize well to new data. MRMR’s approach to feature selection leads to a model that is easier to interpret. With fewer, less correlated features, it’s easier to understand the individual impact of each predictor on the target variable. This clear interpretation is often lost in models plagued by multicollinearity, where it is difficult to disentangle the effects of correlated predictors. By carefully selecting features that maximize relevance to the target and minimize redundancy, MRMR helps create a model that is not only simpler but also more predictive. This optimization is particularly beneficial in high-dimensional datasets, where the risk of multicollinearity is higher. Sun et al. (2023) used a dual drone collaborative approach to monitor late blight and spatial distribution of potatoes in a timely and efficient manner. By integrating a vegetation index from a multispectral UAV, a texture index from an RGB drone, and an estimated crown cover trait, they combined a relief-MRMR technique with machine learning modeling algorithms to monitor the late mottling of potatoes. An et al. (2020) used machine learning to estimate the chlorophyll content of rice from hyperspectral data. Machine learning models have shown accuracy and effectiveness for mapping weekly pan evaporation which is essential for agricultural water management (Vishwakarma et al., 2024).

#### 2.4.2 Robust linear regression

Robust linear regression is a statistical technique used to model the relationship between a dependent variable and one or more independent variables. It is designed to address the potential impact of outliers or influential data points that can significantly affect the results of traditional linear regression models. In conventional linear regression, the model aims to minimize the sum of squared differences between observed and predicted values. However, this approach can be highly sensitive to outliers, which can lead to biased parameter estimates and inaccurate forecasts. Robust linear regression uses alternative estimation methods that are less affected by outliers. A robust linear regression model is particularly useful in situations where the data may contain outliers, errors, or deviations from the underlying assumptions of classical linear regression. It provides more reliable parameter estimates and forecasts and provides greater robustness to the effects of extreme observations (Maronna et al., 2006; Rousseeuw and Leroy, 2005; Venables and Ripley, 2002). The Huber loss function is commonly used in robust linear regression. The formula for the Huber loss function is as follows (Equation 13):


(15) $$\text{Lδ}(\text{y},\text{ f}(\text{x}))=\{\begin{array}{l} \frac{1}{2}{\left(y.f\left(x\right)\right)}^{2}\;for\;\left|y-f\left(x\right)\right|\leq \delta \\ \delta *\left(\left|y-f\left(x\right)\right|-\frac{1}{2}\text{δ}\right)\;\;\;\;otherwise. \end{array}$$

where:

Lδ is the Huber loss function.

y is the observed (actual) value.

f(x) is the predicted value.

δ is a tuning parameter that determines the point at which the loss function transitions from quadratic to linear behaviour.

The Huber loss can be conceptualized as the result of convolving the absolute value function with the rectangular function, which is then scaled and translated. This convolution process effectively “smoothens out” the sharp corner that the absolute value function has at the origin. The Huber loss function is a hybrid between the Mean Squared Error (MSE) and Mean Absolute Error (MAE). It is quadratic for small residuals (errors) and linear for large residuals. This duality allows it to combine the advantages of both MSE and MAE. MSE is sensitive to outliers because the error is squared, which can disproportionately penalize large errors. MAE, on the other hand, is more robust to outliers but may be less sensitive to smaller errors. The Huber loss offers a compromise by treating small errors like MSE (more sensitive) and large errors like MAE (less sensitive), making it effective when the data contains outliers. It doesn’t over-penalize large deviations as much as MSE, preventing the model from being overly influenced by a few extreme values. This can lead to better generalization on unseen data, particularly when the dataset has noise or outliers. Unlike MAE, which can lead to non-differentiable points, the Huber loss is smooth and differentiable everywhere.

#### 2.4.3 Stepwise linear regression

**Figure 2:**
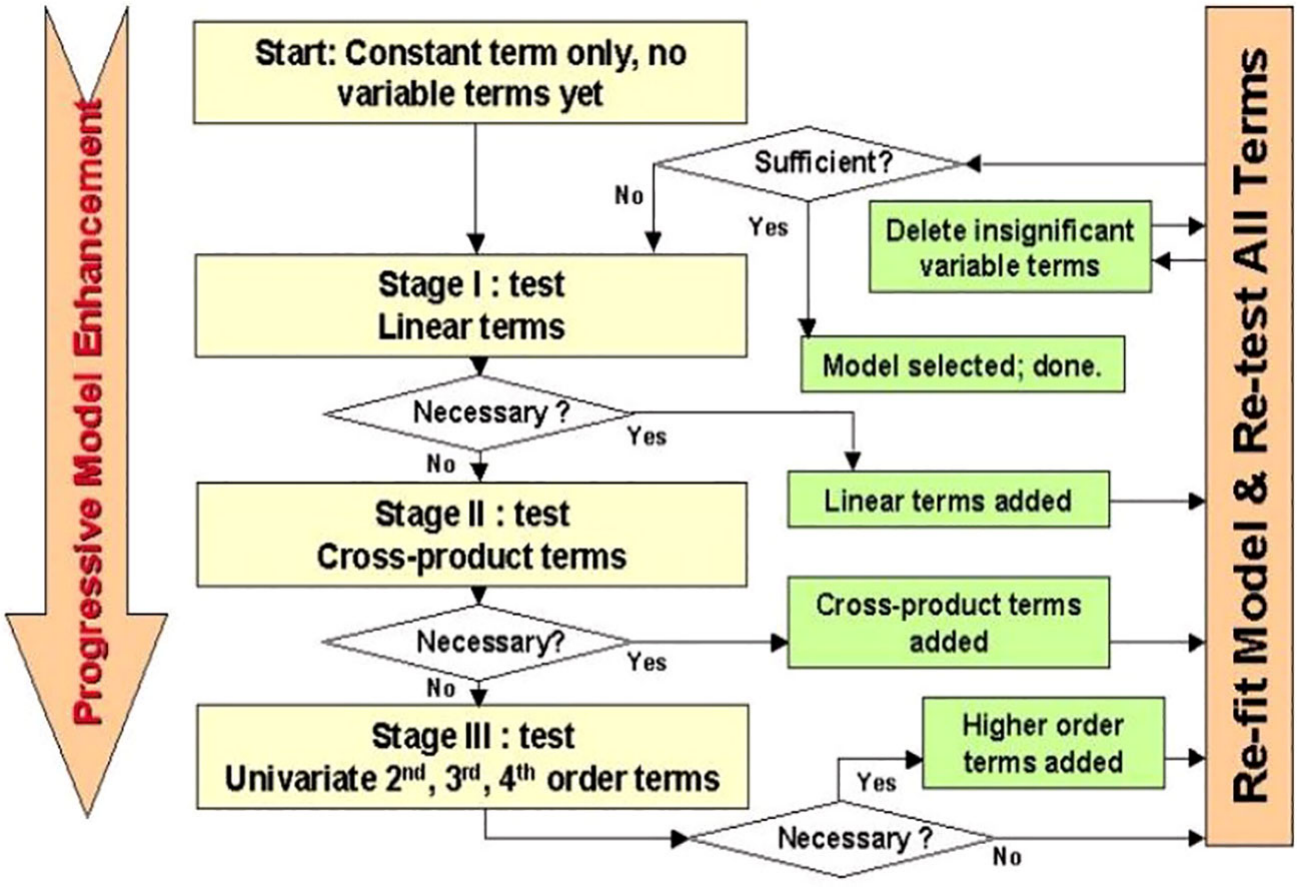
Steps of the Stepwise linear regression (Kim et al., 2012).

Stepwise linear regression is a statistical method used to identify the most significant independent variables to include in a linear regression model. It is a variable selection technique that constructs a regression model by adding or removing variables one by one according to certain criteria. Common entry and exit criteria for selecting variables include p-values, adjusted R-squared, AIC (Akaike information criterion), BIC (Bayesian information criterion) or other model selection criteria. It is essential to understand the implications and assumptions of the criteria chosen (Mendenhall and Sincich, 1995; Kutner et al., 2004; Gareth et al., 2013). The primary strategies employed in stepwise regression include (1) Forward Selection: Begin with an empty model, assessing the addition of each variable based on a selected model fit criterion. Incorporate the variable (if any) that contributes the most statistically significant enhancement to the model fit. Iterate this process until the inclusion of additional variables no longer yields a statistically significant improvement. (2) Backward Elimination: Start with all potential variables included in the model. Evaluate the deletion of each variable using a designated model fit criterion. Remove the variable (if any) whose exclusion results in the least statistically significant deterioration of the model fit. Continue this process until further removal of variables would lead to a statistically significant loss of fit. (3) Bidirectional Elimination: Merge aspects of both forward selection and backward elimination. At each step, assess variables for inclusion or exclusion based on specific criteria. Implement a comprehensive approach by iteratively refining the model through variable additions or deletions. These stepwise regression techniques aim to systematically determine the optimal set of predictors for the model, balancing model complexity and statistical significance (**Figure 2**).

#### 2.4.4 Support vector machines

**Figure 3:**
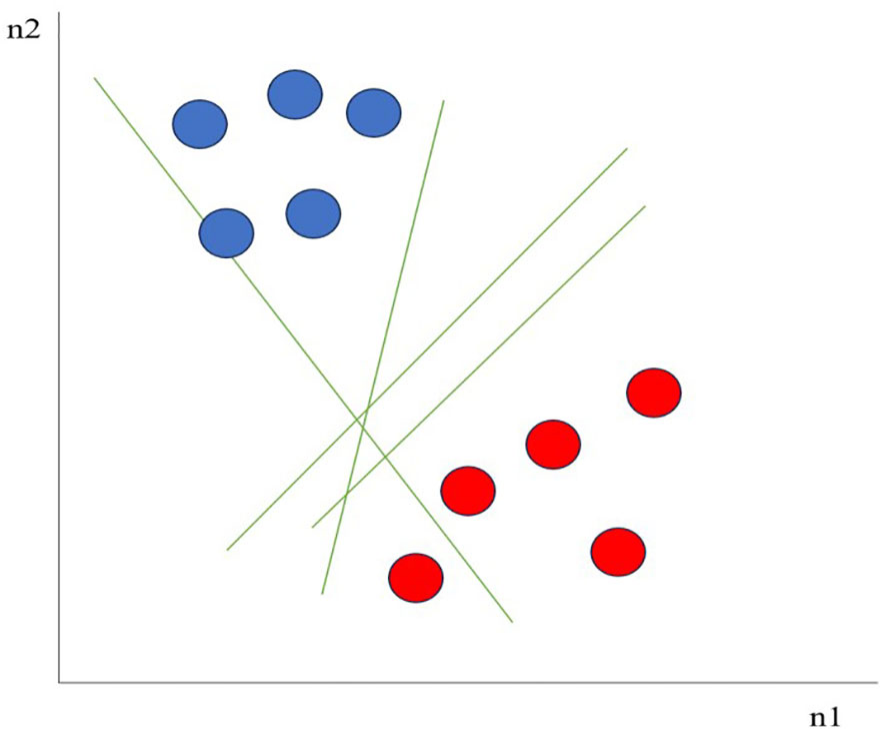
Support Vector Machines.

Support Vector Machines (SVMs) are a type of supervised machine learning algorithm used for classification and regression tasks. They are particularly efficient in high-dimensional spaces and are well-suited for scenarios where there is a clear distinction between classes (Elbeltagi et al., 2022). For example, as seen in **Figure 3** considering two independent variables n1, and n2, and one dependent variable which is either a blue circle or a red circle, there are multiple lines (hyperplanes) that could segregate data points or do a classification between red and blue circles. SVMs aim to find a hyperplane in an n-dimensional space (where n is the number of features) that best separates the data into two classes. SVMs can be used for both classification and regression tasks. When used for classification, SVMs are often referred to as Support Vector Classification (SVC). For regression tasks, they are known as Support Vector Regression (SVR). SVMs are inherently binary classifiers. To extend them to multiclass problems, they usually use strategies such as one-vs-one or one-vs-all. When working with SVMs, it is important to carefully select the kernel and tune the parameters to achieve the best performance on a given task (Shawe-Taylor and Cristianini, 2004) (**Figure 3**).

#### 2.4.5 The gaussian kernel

The Gaussian kernel is often associated with support vector machines (SVM), especially in the context of nonlinear classification problems. The Gaussian kernel is a popular choice for SVMs, especially when dealing with non-linear relationships between features and the target variable. The kernel function takes two data points as input and calculates the similarity between them in a high-dimensional space (Scholkopf and Smola, 2002). The Gaussian kernel is defined as (Equation 14):


(16) $$K\left(x,y\right)=exp\left(-\frac{{\left\|x-y\right\|}^{2}}{2\sigma^{2}}\right)$$

where:

K (x,y) is the value of the Gaussian kernel for points x and y ||x−y|| represents the Euclidean distance between points x and y σ (sigma) is the bandwidth or spread parameter of the kernel, controlling the width of the bell curve.

In the context of SVM, the Gaussian kernel allows SVM to operate in a higher-dimensional space without explicitly calculating the transformations. It captures the similarity between data points in the original feature space, and the transformation induced by the kernel corresponds to projecting the data into a higher-dimensional space where a linear decision boundary may be more easily found.

#### 2.4.6 The matern 5/2 gaussian process regression

The Matern 5/2 Gaussian Process Regression function is capable of modeling relatively smooth functions while allowing a certain level of flexibility in capturing complex patterns. In Gaussian process regression, the Matern 5/2 kernel is used to determine the covariance (or similarity) between different points in the input space. The GP regression model essentially models the distribution between functions, and the choice of kernel determines the characteristics of these functions (Duvenaud et al., 2011). The Matérn 5/2 covariance function is a specific member of the Matérn family and is defined as follows (Equation 15):


(17) $$k_{Matern\;5/2}\;\left(x,x^{\prime }\right)=\;\text{σ}\;\left(1+\;\frac{\sqrt{5\text{r}}}{\text{l}}+\;\frac{5{\text{r}}^{2}}{3{\text{l}}^{2}}\right)exp\left(-\frac{\sqrt{5\text{r}}}{\text{l}}\right)$$

where:

$k_{Matern\;5/2}\;\left(x,x^{\prime }\right)$ is the covariance between points x and x’ according to the Matérn 5/2 kernel

σ is the variance parameter, representing the vertical variation of the function

l  is the length scale parameter, controlling the length of the wiggles in the function

r is the distance (or time difference, depending on the context) between two points

#### 2.4.7 The trilayered neural network

**Figure 4:**
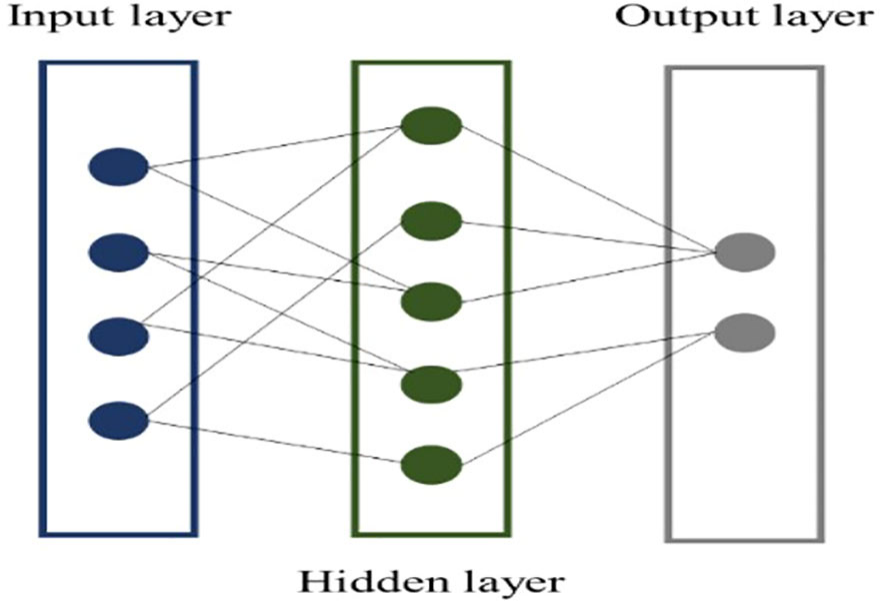
Steps of the trilayered neural network.

The Trilayered Neural Network generally refers to a neural network architecture with three layers: an input layer, a hidden layer, and an output layer (Pande et al., 2022). It is the simplest form of neural network that can learn non-linear representations of data. The layers are connected by weights and each layer consists of nodes or neurons. The structure is often represented as (input layer) - (hidden layer) - (output layer) (**Figure 4**). Hyper-parameters used for the developed machine learning models for estimating crop chlorophyll content are presented in **Table 1**.

**Table 1:** Hyper-parameters used for the developed machine learning models for estimating crop chlorophyll content.

Preset	Hyper-parameters
Robust Linear	Terms: Linear; Robust option: On
Stepwise Linear	Initial terms: Linear; Upper bound on terms: Interactions; Maximum number of steps: 1000
SVM	Kernel function: Quadratic; Kernel scale: Automatic; Box constraint: Automatic; Epsilon: Auto; Standardize data: Yes
Fine Gaussian SVM	Kernel function: Gaussian; Kernel scale: 0.83; Box constraint: Automatic; Epsilon: Auto; Standardize data: Yes
Matern 5/2 GPR	Basis function: Constant; Kernel function: Matern 5/2; Use isotropic kernel: Yes; Kernel scale: Automatic; Signal standard deviation: Automatic; Sigma: Automatic; Standardize data: Yes; Optimize numeric parameters: Yes
Trilayered Neural Network	Number of fully connected layers: 3; First layer size: 20; Second layer size: 20; Third layer size: 20; Activation: ReLU; Iteration limit: 1000; Regularization strength (Lambda): 0; Standardize data: Yes

## 3 Results

### 3.1 Spectral characteristics of maize

**Figure 5:**
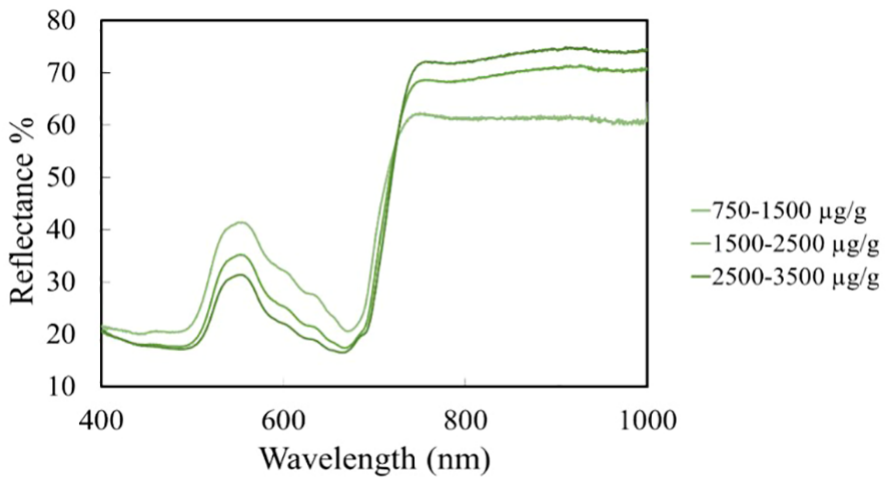
Maize leaf reflectance % values.

Chlorophyll content reflectance profiles were evaluated in the wavelength range of 400-1000 nm. The lowest chlorophyll content was 796.64 µg/g and the highest chlorophyll content was 3257.03 µg/g. It was observed that leaves with high chlorophyll content showed a reflectance value between 20-30%, which shows an increasing reflectance with decreasing chlorophyll values. At low chlorophyll values of 600-1000 µg/g, a reflectance value of 45-48% was observed. The maximum reflectance of carotenoids was measured in the 520-580 nm wavelength range, which gave a low reflectance value of around 31% at high chlorophyll content. As the chlorophyll content decreases, the reflectance value increases proportionally for the carotenoid content. Carotenoids at low chlorophyll content interval values gave a reflectance value of 46%. Plant stress can be detected with high reflectance values in the wavelength range 500-700 nm (**Figure 5**). Based on the structural properties of the leaf, most of the energy is transmitted and reflected, which creates a high near-infrared (NIR) curve. The red rim, which is located between the red and NIR bands during a sharp rise in reflectance, is used to detect plant stress and is more closely associated with pigments (Kior et al., 2021) Vegetation indices are derived mainly from red and NIR band reflectance data, numerical measurements that measure biomass or the progress of vegetation status based on the spectral characteristics of vegetation (Roman and Ursu, 2016).

**Figure 6:**
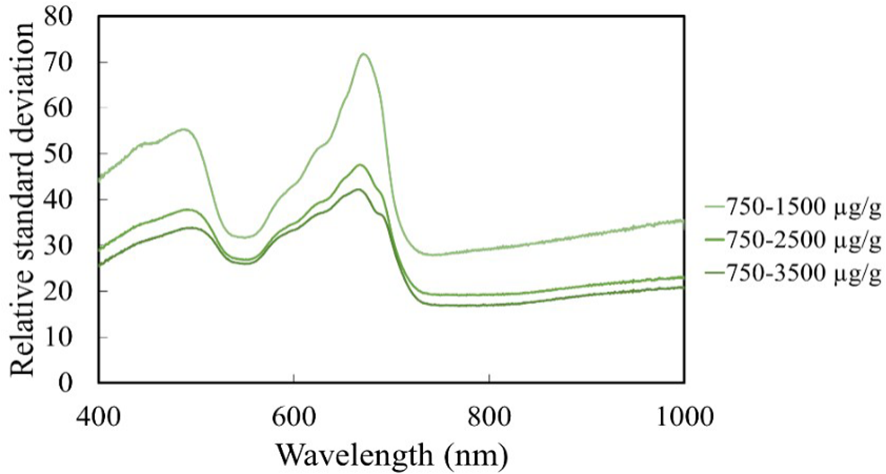
Relative standard deviation values of the reflectance of maize.

To further investigate the spectral characteristics of the leaf samples, the relative standard deviation values of the reflectance (%) data were divided into three groups based on chlorophyll content (750-1500 µg/g, 750-2500 µg/g, 750-3500 µg/g). Low standard deviation (up to 520 nm ± 30 nm) was observed in the low chlorophyll groups. The standard deviation of reflectance increased with chlorophyll content. Therefore, this range may be suitable for plant maturity assays. The peak of the standard deviation is prominent at 670 nm for high chlorophyll content due to the absorption characteristics of chlorophyll measured in this wavelength range. It is observed that the standard deviation of reflectance values calculated in the wavelength ranges 550 nm, 670 nm, and 700 nm is pigment sensitive. This sensitivity decreases due to an increase in absorbance with increasing carotenoid content. Thus, this spectral characteristic disappears with increasing carotenoid content (**Figure 6**). Nagy et al. (2022) and Zur et al. (2000) have confirmed the variation in the pigment content of leaves in their studies and reached similar conclusions to the present study.

### 3.2 Calibration of the models

**Figure 7:**
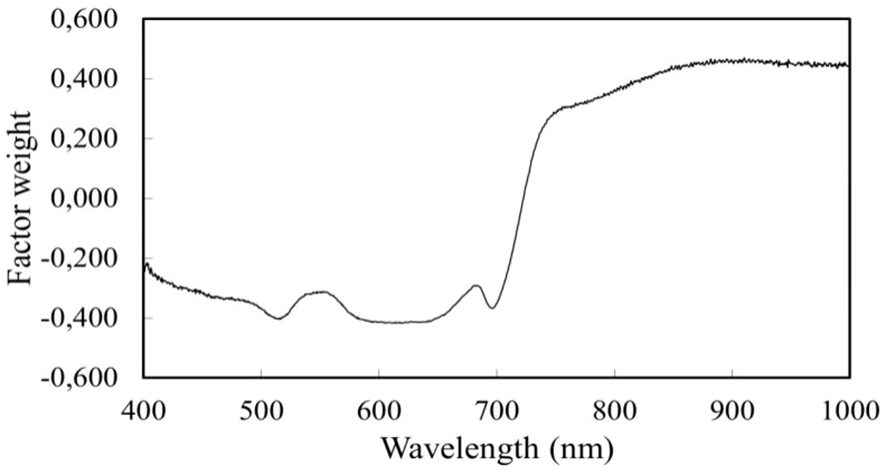
Factor analysis of spectral values.

The PCA resulted in five principal components. Based on the factor weights of the first component, the two largest variances of the reflectance are observed in the wavelength range 516 and 551 nm. There were two minima in the factor weight, of which the 763 nm range was used together with the 516 and 551 nm ranges to construct the chlorophyll estimator indices (**Figure 7**).

Three indices were created based on the results of principal component analysis and standard deviation:


$${\text{CHLI}}_{1}=\;\left(\lambda_{551}+\lambda_{763}\right)/\left(\lambda_{763}-\lambda_{551}\right)$$

$${\text{CHLI}}_{2}=\;\left(\lambda_{763}-\lambda_{516}\right)/\lambda_{551}$$

$${\text{CHLI}}_{3}=\;\left(\lambda_{516}+\lambda_{551}\right)/\lambda_{763}$$

This was based on linear regression with a medium regression value R=-0.76 (p=0.000). The model showed a medium correlation with R=0.72 (p=0.000). The model also showed a medium regression with R=0.77 (p=0.000). In addition to the developed models, for comparison, VIs already used in practice are also considered (**Table 2**). In the case of NDVI, R=0.54, during the REP index, as the leaf point with the maximum slope between the red and near-infrared wavelengths of the plant reflectance spectrum, we obtained R=0.81. During the calculation of the Red Edge Normalized Difference Vegetation Index, we obtained R = 0.80. During the Modified Red Edge Simple Ratio Index, we obtained R=0.47. When calculating the Modified Red Edge Normalized Difference Vegetation Index, R=0.81 was obtained, and during the Photochemical Reflectance Index, a low value of R=0.12 was obtained. When calculating the Modified Chlorophyll Absorption Ratio Index, we obtained a value of R=-0.74. The calculation of the Vogelmann red-edge index 2 gave a value of R=-0.76 (**Table 3**).

**Table 2:** Descriptive statistics of chlorophyll and vegetation indices.

Variable	Mean	StDev	Minimum	Q1	Median	Q3	Maximum	Skewness	Kurtosis	MSSD
NDVI	0.31577	0.04733	0.14267	0.29103	0.31703	0.34265	0.45749	-0.37	1.11	0.00159
REP	715.59	6.02	675.81	714.90	717.86	718.91	722.41	-2.56	8.09	6.38
NDVI705	0.33656	0.07733	0.06876	0.31328	0.35712	0.38414	0.49674	-1.29	1.44	0.00160
mSR705	0.93222	0.17080	0.35818	0.82021	0.93350	1.01252	1.68291	0.52	2.02	0.02445
mNDVI705	0.55517	0.13735	0.10424	0.49871	0.60995	0.65088	0.74525	-1.28	0.73	0.00458
PRI	0.014405	0.007429	-0.025879	0.010403	0.013689	0.017459	0.044902	-0.19	4.55	0.000044
MCARI	19.572	14.293	5.832	10.268	13.440	22.880	80.894	1.75	2.40	74.826
VREI2	-0.05873	0.02577	-0.12875	-0.07693	-0.06576	-0.03974	0.00005	0.50	-0.60	0.00025
Index1	2.7650	1.2309	1.7205	2.1998	2.3600	2.7112	13.5331	3.79	19.15	0.3090
Index2	1.6067	0.4269	0.3827	1.4045	1.6653	1.8513	3.0671	-0.31	0.82	0.0848
Index3	-0.09868	0.04824	-0.25874	-0.13622	-0.07523	-0.06444	-0.04863	-1.12	-0.13	0.00077
Chlorophyll	2479.8	632.5	321.1	2188.8	2640.4	2937.6	3713.1	-1.00	0.57	114.2

*mean of the squared successive differences (MSSD).

**Table 3:** Statistical results.

Index	R^2^	RMSE	MAE	MBD	MSPE
CHLI_1_	0.605	393.580	310.887	-7.651	58.537
CHLI_2_	0.518	438.496	345.437	0.0215	0.000463
CHLI_3_	0.641	376.743	297.114	3.960	15.68362
NDVI	0.293	531.259	422.817	-0.00153	2.36E-06
REP	0.654	371.573	298.750	-0.513	0.263
NDVI_705_	0.645	375.991	297.805	-0.0113	0.000128
mSR_705_	0.222	557.331	458.352	0.0294	0.000866
mNDVI_705_	0.650	373.677	297.970	-0.0123	0.000152
PRI	0.015	626.971	496.962	0.0249	0.00062
MCARI	0.544	426.579	341.269	0.00672	4.52E-05
VREI2	0.645	376.081	297.759	0.0117	0.000137

### 3.3 Validation of the new models

**Figure 8:**
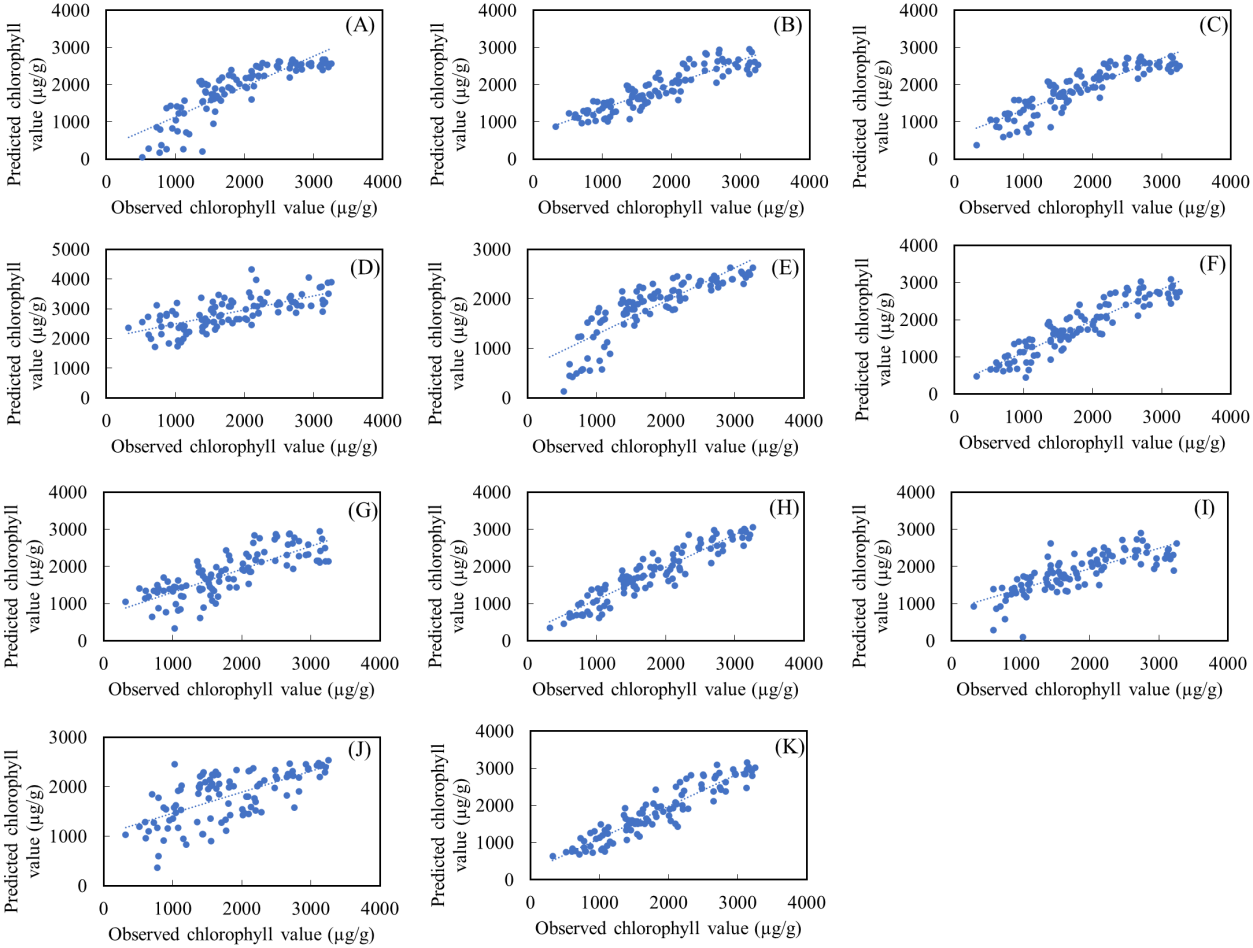
Actual versus predicted values of chlorophyll for the linear regression models during the training period **(A)** Validation of CHL_1_, **(B)** Validation of CHL_2_, **(C)** Validation of CHL_3_, **(D)** Validation of NDVI, **(E)** Validation of REP, **(F)** Validation of NDVI _70_5, **(G)** Validation of mSR_705_, **(H)** Validation of mNDVI_705_, **(I)** Validation of PRI, **(J)** Validation of MCARI, **(K)** Validation of VREI2.

R^2^ is the proportion of variance explained, which shows how well the model fits the data. The closer the value is to 1, the better the model fits the data. The RMSE and MAE values are indicators of the accuracy of the model’s predictions, where smaller values are better. Mean Bias Deviation (MBD) is a statistical measure used to assess the accuracy of a model’s predictions compared to actual observations. Mean Squared Prediction Error (MSPE) is a statistical measure used to evaluate the accuracy of a predictive model. It calculates the average of the squared differences between the predicted values and the actual observed values in a test dataset. Based on these, the REP (R^2^ = 0.65, RMSE=371.57, MAE=298.75, MBD= -0.513, MSPE=0.263); NDVI_705_ (R^2^ = 0.65, RMSE=376.00, MSE=297.81, MBD= -0.0113, MSPE=0.000128); mNDVI_705_ (R^2^ = 0.65, RMSE= 373.68, MAE=297.97, MBD= -0.0123, MSPE=0.000152); VREI2 (R^2^ = 0.65, RMSE=376.08, MAE=297.76, MBD= 0.0117, MSPE=0.000137) and CHL_3_ (R^2^ = 0.64, RMSE=376.74, MAE= 297.12, MBD= 3.960, MSPE=58.537) indices appear to perform best on the datasets under consideration. (**Figure 8**) (**Table 3**).

### 3.4 Training and testing results of machine learning models

A strong positive correlation is observed between NDVI and REP with a correlation of 0.66 and between NDVI and NDVI_705_ with a correlation of 0.76. In both cases, a strong positive relationship is indicated, i.e. when one variable increases, the other increases. The correlation between Index_2_ and Index_3_ is also high at 0.94, suggesting that these variables are closely related. A strong negative correlation is observed between MCARI and mNDVI_705_ with a correlation of -0.91, and between MCARI and VREI2 with a correlation of -0.92. In both cases, they show a strong negative relationship, i.e. when one variable increases, the other decreases. Medium-strength correlations are observed for the correlation between Index1 and Index_3_ of 0.71 and the correlation between Index_2_ and Index_3_ of -0.80. These represent medium-strength relationships. Weak correlations are observed between NDVI and PRI with a correlation of 0.26, and between NDVI_705_ and PRI with a correlation of 0.20. These indicate weaker relationships. There are moderate (e.g. 0.80 between chlorophyll and NDVI_705_), strong positive (e.g. 0.81 between chlorophyll and REP), and strong negative (e.g. -0.74 between chlorophyll and MCARI) correlations between chlorophyll and the other variables (**Table 4**). The tolerance metric reveals how effectively a specific variable can be forecasted by other variables within the model. Notably, the REP variable exhibits an exceptionally low tolerance (0.01), suggesting a substantial linear interdependence with other variables. Similarly, low tolerances are observed for the rest of the indices indicating potential strong dependencies, except for PRI and NDVI. Elevated VIF values imply a linear dependence on other variables. Remarkably high VIFs for the REP and VREI2 variables (71.83 and 24.77, respectively) point to a pronounced linear dependence on other variables. Additionally, the Index2, Index3, and mSR705 variables exhibit high VIFs, suggesting substantial dependencies. These findings highlight significant correlations among certain variables in the model, indicating a multicollinearity. The PRI shows 0.47 tolerance and 2.11 VIF values, which exceeds the thresholds for both indicators, therefore PRI shows no collinearity, and thus there is a high probability for not using to develop good estimator models. In the case of NDVI, the VIF value just slightly exceeds the threshold (5) (**Table 4**).

**Table 4:** Correlation matrix of spectral indices and chlorophyll content (A), Multicollinearity statistics analysis of spectral indices (B).

	NDVI	REP	NDVI705	mSR705	mNDVI705	PRI	MCARI	VREI2	Index1	Index2	Index3
Correlation Matrix (A)											
REP	0.657										
NDVI705	0.759	0.915									
mSR705	0.776	0.501	0.743								
mNDVI705	0.590	0.939	0.929	0.448							
PRI	0.261	0.148	0.200	0.440	0.019						
MCARI	-0.456	-0.867	-0.803	-0.283	-0.910	0.105					
VREI2	-0.692	-0.849	-0.924	-0.582	-0.921	0.062	0.823				
Index1	-0.655	-0.944	-0.883	-0.613	-0.833	-0.307	0.707	0.744			
Index2	0.786	0.795	0.949	0.861	0.799	0.235	-0.678	-0.875	-0.804		
Index3	0.465	0.860	0.830	0.314	0.938	-0.137	-0.922	-0.906	-0.718	0.738	
Chlorophyll	0.541	0.809	0.804	0.473	0.806	0.124	-0.737	-0.760	-0.764	0.721	0.770
Multicollinearity statistics analysis (B)											
Tolerance	0.198	0.014	0.003	0.010	0.006	0.474	0.072	0.040	0.031	0.014	0.026
VIF	5.056	71.827	384.170	100.589	165.484	2.108	13.802	24.774	32.045	69.603	38.203

The values in the MRMR (Minimum Redundancy Maximum Relevance) database are used to indicate the relevance of each feature to the model. The mNDVI_705_ is the most important feature of the model. Its high importance value shows that this feature can make a significant contribution to the model performance. The second most important feature is mSR_705_. It also has a significant contribution to the model. Index3 is also an important characteristic, with a rather high value, indicating that it has important information for the target variable. The NDVI_705_ characteristic also has a high importance value. The VREI2 attribute has a lower importance value but still contributes significantly to the model. Index_2_ is also important but with a lower importance value. The MCARI characteristic also contributes to the model. The REP characteristic is still important but less significant than the others. The Index_1_ characteristic has a lower importance value. The NDVI characteristic has a relatively lower importance value. The PRI attribute has no importance value (0), indicating that it is of no relevance to the model from a current perspective (**Table 5**).

**Table 5:** Results of the MRMR.

Features	Importance
mNDVI705	0.477
mSR705	0.414
Index3	0.394
NDVI705	0.381
VREI2	0.350
Index2	0.345
MCARI	0.341
REP	0.306
Index1	0.271
NDVI	0.244
PRI	0

The Robust Linear Regression (R^2^ = 0.67) had RMSE=360.39 µg/g, MAE=283.62 µg/g, MBD=21.539 µg/g, MSPE= 463.959 µg/g. An R^2^ value of 0.67 indicates that the model is explanatory and fits your data reasonably well. The RMSE and MAE values are moderately low, suggesting that the model performs well overall. Stepwise Linear Regression (R^2^ = 0.70) RMSE=343.76 µg/g, MAE=267.89 µg/g, MBD=38.597 µg/g, MSPE= 148.757µg/g the model has an even higher R^2^ value and lower RMSE and MAE values, which generally means better fit and prediction. During SVM (R^2^ = 0.67), RMSE=359.65 µg/g, MAE=280.74 µg/g, MBD=23.2743µg/g, MSPE= 541.695 µg/g. The SVM model also performs well, like Robust Linear Regression, but with a slightly higher R^2^ value. Fine Gaussian SVM (R^2^ = 0.48) RMSE=467.54 µg/g, and MAE=340.28 µg/g, the model has a lower R^2^ value and higher RMSE and MAE values, which may indicate that it is less well fitted to your data. Matern 5/2 Gaussian Process Regression (R^2^ = 0.42) RMSE=338.46 µg/g, and MAE=264.30 µg/g, this model fits your data relatively well, with high R^2^ values and lower RMSE, MAE values. The Trilayered Neural Network (R^2^ = 0.38) RMSE=493.44 µg/g, MAE=378.42 µg/g, MBD= -70.253 µg/g, MSPE= 630.172 µg/g the model has a low R^2^ value and higher RMSE, MAE values, which may indicate that the modeling is less efficient in this case (**Table 6**; **Figure 9**). The Robust Linear Regression (R^2^ = 0.748) had RMSE=324.17 µg/g, MAE=262.26 µg/g, MBD=-79.625 µg/g, MSPE= 493.569 µg/g. An R^2^ value of 0.748 indicates that the model is explanatory and fits your data well. The RMSE and MAE values are moderately low, suggesting that the model provides a good fit and prediction. Stepwise Linear Regression (R^2^ = 0.75) RMSE=322.10 µg/g, MAE=247.11 µg/g, MBD= 10.4014 µg/g, MSPE= 108.186 µg/g the model has an even higher R^2^ value and lower RMSE and MAE values, which generally means better fit and prediction. The SVM (R^2^ = 0.75), RMSE=325.74 µg/g, and MAE=239.86 µg/g, this model also performs well, like Robust Linear Regression, but with a slightly lower R^2^. Fine Gaussian SVM (R^2^ = 0.48) RMSE=467.54 µg/g, and MAE=340.28 µg/g, the model has a lower R^2^ value and higher RMSE, MAE values, which may indicate that it is less well fitted to your data. Matern 5/2 Gaussian Process Regression (R^2^ = 0.79) RMSE=296.37 µg/g, MAE=237.12 µg/g, MBD= 2.195 µg/g, MSPE= 4.820 µg/g the model fits your data relatively well, with high R^2^ values and lower RMSE, MAE values. The Trilayered Neural Network (R^2^ = 0.58) RMSE=419.03 µg/g, MAE=335.42 µg/g, MBD= -29.545 µg/g, MSPE= 872.929 µg/g the model has a low R^2^ value and higher RMSE, MAE values, which may indicate that the modeling is less efficient in this case. Almost identical accuracy values were observed for the models created, with the Matern 5/2 Gaussian process regression giving the most accurate prediction value when comparing the results (**Figures 9A, B**, **10**) (**Table 6**).

**Table 6:** Statistical performance evaluation for the developed ML models in chlorophyll estimation during the training and testing stages.

Model Type	Training stage					Testing stage				
	R^2^	RMSE	MAE	MBD	MSPE	R^2^	RMSE	MAE	MBD	MSPE
Robust Linear Regression	0.670	360.389	283.623	21.539	463.959	0.748	324.178	262.269	-70.253	493.569
Stepwise Linear Regression	0.700	343.758	267.887	38.597	148.757	0.751	322.109	247.110	10.4014	108.186
SVM	0.672	359.647	280.744	23.2743	541.695	0.745	325.749	239.868	26.610	70.095
Fine Gaussian SVM	0.423	477.135	364.949	-15.9187	253.405	0.475	467.544	340.283	30.456	76.424
Matern 5/2 Gaussian Process Regression	0.709	338.461	264.297	-94.253	883.694	0.789	296.378	237.128	2.195	4.820
Trilayered Neural Network	0.383	493.444	378.418	-79.625	630.172	0.578	419.030	335.426	-29.545	872.929

**Figure 9A:**
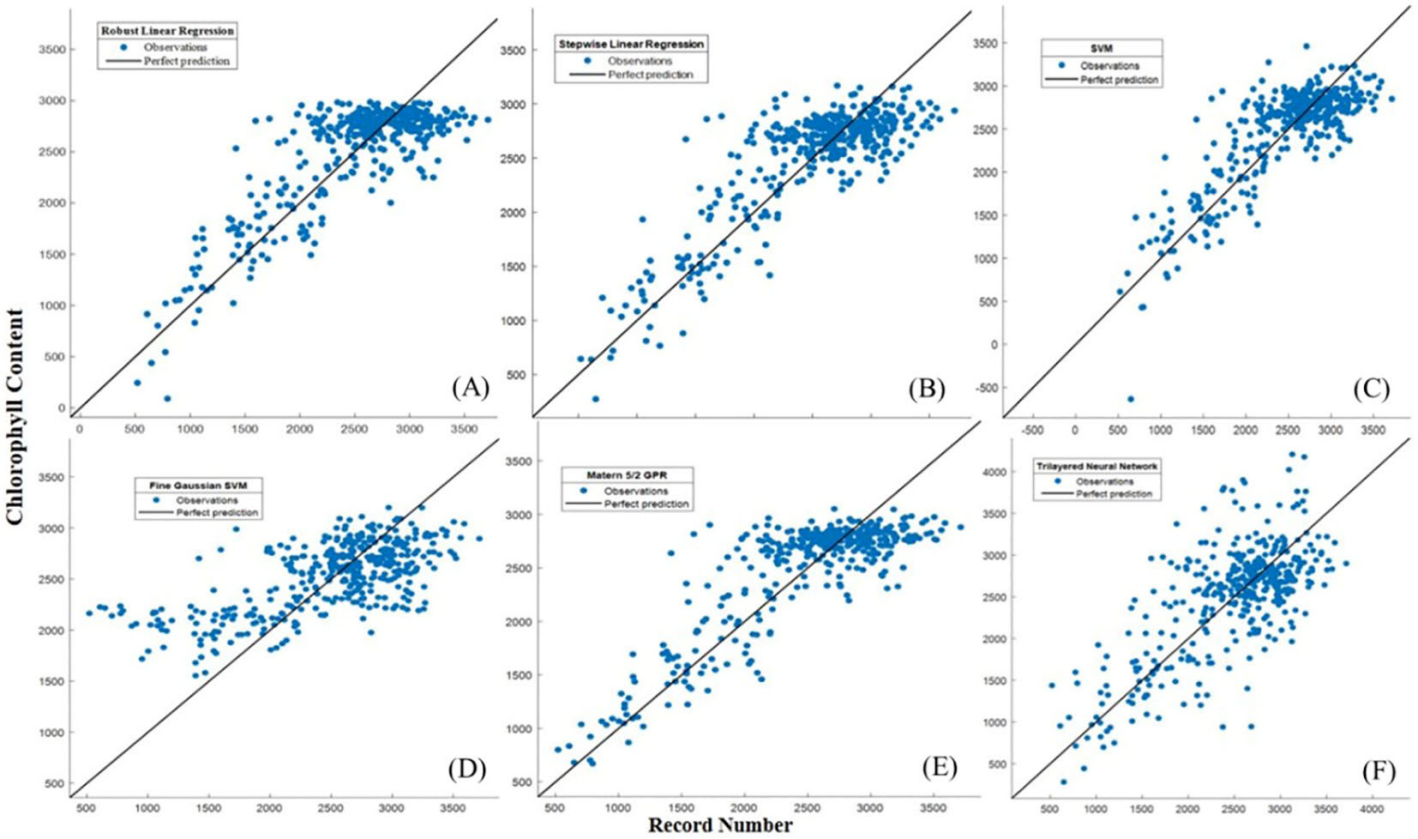
Actual versus predicted values of chlorophyll for the ML developed models during the testing period **(A)** Robust linear regression, **(B)** Stepwise linear regression, **(C)** SVM, **(D)** Fine Gaussian SVM, **(E)** Matern 5/2 GPR, **(F)** Trilayered Neural Network.

**Figure 9B:**
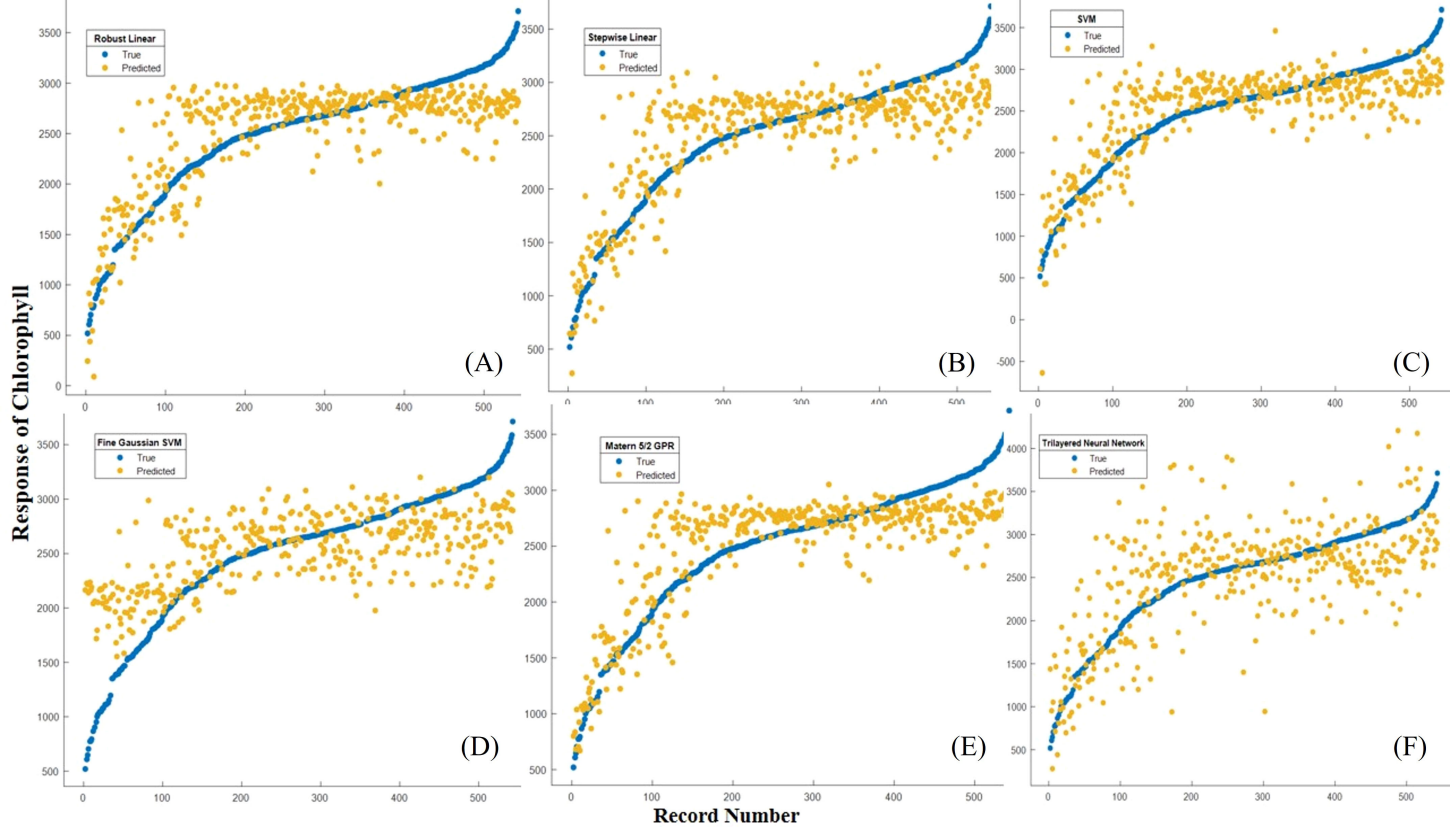
Record number versus chlorophyll for the ML-developed models during the training period **(A)** Robust linear regression, **(B)** Stepwise linear regression, **(C)** SVM, **(D)** Fine Gaussian SVM, **(E)** Matern 5/2 GPR, **(F)** Trilayered Neural Network.

**Figure 10:**
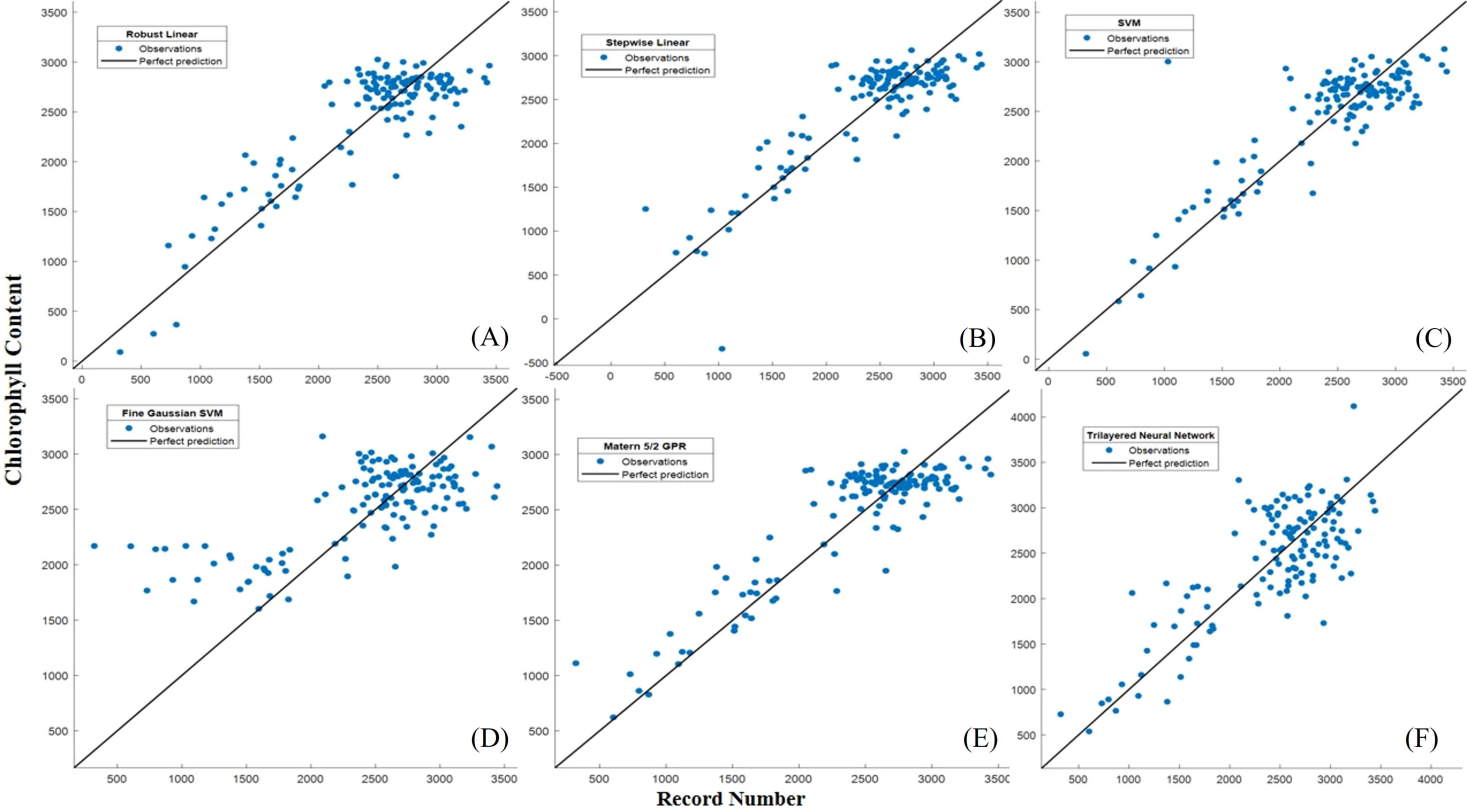
**(A)** Actual versus predicted values of chlorophyll for the ML developed models during the training period **(A)** Robust linear regression, **(B)** Stepwise linear regression, **(C)** SVM, **(D)** Fine Gaussian SVM, **(E)** Matern 5/2 GPR, **(F)** Trilayered Neural Network.

## 4 Discussion

There have been several research in the literature that uses machine learning to estimate the chlorophyll content of maize plants. However, few researchers have examined the effectiveness of various machine-learning algorithms in assessing maize’s chlorophyll content (Berry and Bjorkman, 1980; Munson and Caruana, 2009). Furthermore, a limited number of studies have provided a thorough description of the machine learning parameter optimization process through training and validation. In the present research, six machine learning models (RL, SR, SVMs, FG-SVM, MG-PR, and TNN) were optimized and developed using in hyperspectral indices, employing the MRMR algorithm and PCA.

In this study, we observed the significance of standard deviation as a crucial feature, likely reflecting seasonal patterns in the chlorophyll target variable. It helped to assess the variability, accuracy, and precision of the predictions. Seasonal patterns play a vital role in chlorophyll content, driven by changes in temperature, light, and plant growth cycle. For example, during the spring and summer, longer daylight hours and warmer temperatures promote photosynthesis, leading to higher chlorophyll content (Berry and Bjorkman, 1980). Moreover, the study conducted a comparison of the performances of three CHLI indices created (**Table 3**) from PCA and various vegetation indices used in practice. The following insights were based on the selected features and the prediction results obtained from these indices. PCA is commonly used as a data pre-processing step in classic machine learning techniques.

The selection of features is a response aimed at balancing bias and variance within the learning algorithms (Munson and Caruana, 2009). This improves the separation accuracy of biophysical parameters. The MRMR algorithm illustrated that it can be employed to enhance environmental noise filtering, aiding in the differentiation between noise and the specific characteristic of interest by filtering less important indexes such as PRI. Variables such as NDVI_705_, mSR705, mNDVI_705_, Index1, Index2, and Index3 have low tolerance values, indicating potential multicollinearity issues and as such, the need to address them in the regression model to avoid biased coefficient estimates and unstable predictions. However there are additional interpretability techniques like Shapley Additive Explanations (SHAP) or Local Interpretable Model-agnostic Explanations (LIME) to demonstrate which features most influenced the machine learning model predictions (REF), which are planned to implement inb our further sstudies (Ma et al., 2022; Khan et al., 2024).

The Matern 5/2 Gaussian Process Regression (MG-PR) model appears to have the highest R^2^ and relatively lower RMSE and MAE, suggesting it performs well compared to the other models. It demonstrated the highest accuracy in both the training set and validation set. Notably, while the training set result did not surpass the validation set result, it implies that the generalization of MG-PR is relatively good. It’s worth noting that this algorithm typically benefits from a relatively large dataset to mitigate overfitting issues (Manzhos and Ihara, 2023). In this study, the model’s moderate RMSE indicates few anomalies or outliers in the predicted maize chlorophyll content. **Figure 10** illustrates that none of the algorithms succeeded in forecasting extreme values or outliers.

The performance of Robust Linear Regression (RL) ranked as the second-best after Matern 5/2 Gaussian Process Regression (MG-PR). The characteristics of RL’s performance slightly differed from MG-PR; while MG-PR exhibited an excellent goodness-of-fit, it also demonstrated relatively good prediction results. Similar trends were observed in the RMSE and MAE for both RL and MG-PR. Specifically, the MAE increased for both the training set and validation set of MG-PR, and the RMSE increased for both the training set and validation set of RL. In both instances, there was a general decline in performance metrics between the training and validation stages for both models.

Therefore, in comparison to RL, MG-PR exhibited greater generalization and demonstrated stronger stability, as evidenced by its superior performance across various metrics and stages.

From **Tables 4**, **5**, indices like REP, NDVI705, and mNDVI705 demonstrate strong positive correlations with chlorophyll content, making them potentially valuable for estimating chlorophyll levels. On the other hand, MCARI and VREI2 show strong negative correlations, suggesting their potential as indicators of lower chlorophyll content. Indices like CHLI_2_, CHLI_3_, NDVI_705_, mNDVI_705_, and VREI2 show high R^2^ values and lower RMSE and MAE, suggesting their effectiveness in predicting chlorophyll content. NDVI, mSR705, and PRI exhibit moderate performance, while MCARI shows relatively lower predictive capabilities.

The SVM classifier, a kernel-based classification approach, has been successfully used in estimating crop parameters in studies such as Karimi et al. (2008) and Kumar et al. (2015). Behmann et al. (2014) used ordinal SVM (accuracy of 68%) to detect water stress in barley leaves using hyperspectral images. In our study, however, when combined with a fine Gaussian filter, the SVM classifier performs the worst with an accuracy of 42.3%. The evaluations of the six machine learning techniques suggest that the generated CHLI indices serve as promising variables for estimating the chlorophyll content of maize. The predictive results indicate the effectiveness of the selected features (**Table 6**) in estimating the chlorophyll content of maize using machine learning algorithms. The mNDVI_705_ index had the best accuracy at 87.50% followed by VREI2 at 86.90%.

The findings indicate that incorporating TNN, which exhibited the second lowest R^2^ and the highest metrics for chlorophyll content within the dataset, proved less effective in estimating chlorophyll content in contrast to successful crop classification and yield prediction studies (Subbarao et al., 2023; Lagrazon and Tan, 2023) Moreover, these results underscore the effectiveness of employing hyperspectral reflectance combined with machine learning algorithms. This approach not only enables the prediction of chlorophyll content, as previously documented by Singh et al. (2022), but also facilitates the non-destructive estimation of nitrogen (N) content in maize leaves using the PROSPECT-PRO model (Féret et al., 2021).

When compared with the classical vegetation indices used in practice, neither the univariate nor linear approaches achieved the requisite computational performance to surpass the ML models. The improved R^2^, MAE, and RMSE results in the independent validation dataset could be attributed to reduced variability and the absence of extreme values, demonstrating the robust generalization capabilities of the machine learning (ML) models. In contrast, various studies in the ML literature, such as Ciganda et al. (2009), have reported R^2^ values ranging from 0.80 and 0.87 while analyzing longitudinal time series of chlorophyll in maize leaves and canopy respectively. Sudu et al. (2022) had a R^2^ using 0.81, 0.42, 0.65, and 0.82 using PLSR, RF, XGBoost, and the DNN ML. Authors such as Angel and McCabe (2022) employed restraining vs sequential learning models to predict chlorophyll variations, yielding R^2^ results ranging from 0.59 to 0.74. Similarly, Guo et al. (2022) using RF for spatial predictions of chlorophyll using a SPAD device, achieved the highest R^2^ value of R^2^ (0.81) and RMSE (0.14).

In precision agriculture, the evaluation of RMSE, R², and MAE is often relative to the scale and variability of the measured variable. For instance, when predicting crop chlorophyll, an RMSE that is small relative to typical chlorophyll values—such as 5-10% of the mean—is considered acceptable (Croft et al., 2015). In our study, the RMSE values are indeed small relative to the typical chlorophyll values of 796.64 and 3257.03 µg/g, indicating satisfactory performance. Similarly, while R² values closer to 1 are generally preferred in agricultural applications, an R² of 0.7-0.9 is often deemed good for complex biological systems with high variability, such as chlorophyll predictions (Çalışkan et al., 2020). Our results fall within this acceptable range. Likewise, the acceptability of MAE is context-dependent, with values close to or below the typical variation within the dataset considered good. For example, in predicting plant chlorophyll, an MAE of a few μg/g per week is typically acceptable, depending on the crop and growth stage (Prilianti et al., 2020). In our case, the Stepwise LR MAE meets these acceptable thresholds. These diverse performance metrics highlight the variability in different methods’ effectiveness in predicting chlorophyll levels, suggesting adaptability rather than superiority of one method over another. The ML models in this study demonstrated their capacity for generalization and high accuracy, thereby offering promising prospects for further application and refinement.

The main outcome of this study is the introduction of an innovative methodology combining MRMR and hyperspectral indices. Present results rely on active sensoring technique with spectral measurements in standard light conditions. This can facilitate the improvement of such new field portable devices for rapid non-destructive chlorophyll measurement which are measuring the reflectance of the canopy at the identified wavelengths using the best performing ML method. To apply our method, such type of sensor head is required, which can eliminate the environmental background light circumstances, and uses active light source. However standard light conditions can be a limitation as well in such cases where the sensing would be done by passive sensoring techniques. Considering potential field-based applications of the model with open sensor head further sensitivity analysis is valuable exploring the effect of environmental variability (e.g. albedo, irradiance) and even soil conditions on the reflectance and the explored models. I is also has to be considered, that an overreliance on specialized equipment (such as hyperspectral sensors) and complex machine learning models may limit the broader applicability of the method in regions lacking such resources (Srivastava and Chinnasamy, 2021).

## 5 Conclusion

In conclusion, this research offers a new method for precisely estimating crop chlorophyll levels in maize by combining the Minimum Redundancy Maximum Relevance (MRMR) algorithm with hyperspectral indices and sophisticated machine learning algorithms. With an emphasis on real-time monitoring of crop growth status and optimization of nitrogen fertilizer applications, the study intends to address inadequacies in current chlorophyll estimating methods by utilizing extensive hyper-spectral data. The comprehensive character of the methodology is demonstrated by the investigation of many hyperspectral indices, which includes the development of new spectral indices using a laboratory proximal sensor customized to the concentration of chlorophyll in maize leaves. To develop chlorophyll sensitive indices spectral bands were identified by PCA and spectral characteristics. Six complex machine learning models are used, together with the MRMR feature selection method to build intricate correlations between hyperspectral indices and chlorophyll concentration. Based on stringent evaluation measures, including coefficient of determination (R²), mean absolute error (MAE), and root mean square error (RMSE), Using VI with different feature importance the Matern 5/2 Gaussian Process Regression model was found to be the most accurate model for predicting the amounts of chlorophyll in maize. The results show that new spectral indices coupled with other VIs are effective for non-invasive crop chlorophyll estimation using ML algorithms, with great potential for early interventions to mitigate abiotic stress and optimize agricultural operations.

## Data Availability

The raw data supporting the conclusions of this article will be made available by the authors, without undue reservation.
